# Predicting Complicated Appendicitis: What Can Machine Learning Add?

**DOI:** 10.3390/diagnostics16162644

**Published:** 2026-08-19

**Authors:** Mustafa Alper Akay, Ayşe Nur Kübra Kılıç, Ozan Can Tatar, Onursal Varlıklı, Gülşen Ekingen Yıldız

**Affiliations:** 1 Department of Pediatric Surgery, School of Medicine, Kocaeli University, Izmit 41001, Turkey; pedcerr@gmail.com (M.A.A.); aysnrkbr@gmail.com (A.N.K.K.); ovarlikli@gmail.com (O.V.); gulsen.ekingen@kocaeli.edu.tr (G.E.Y.); 2 Department of General Surgery, Kocaeli Sehir Hastanesi, Izmit 41060, Turkey; 3 Department of Biomedical Engineering, Kocaeli University, Izmit 41001, Turkey

**Keywords:** pediatric appendicitis, machine learning, explainable AI

## Abstract

**Background/Objectives**: Early identification of complicated appendicitis in children remains challenging. We developed and internally validated laboratory-based machine-learning models for severity stratification using age and routine admission laboratory data. **Methods**: This retrospective study included 628 children with surgically confirmed appendicitis treated between 2020 and 2024. Complicated appendicitis was defined by operative or pathological evidence of perforation, gangrene, abscess, phlegmon, diffuse peritonitis, or comparable advanced inflammation. Fifteen candidate predictors were evaluated using five prespecified models. Models were tuned in the training set and evaluated once on an isolated test set. Pairwise DeLong comparisons, decision curve analysis, SHAP, and permutation importance were performed. **Results**: Complicated appendicitis occurred in 93 patients (14.8%). The prespecified primary CatBoost model achieved a ROC AUC of 0.867, a precision-recall AUC of 0.677, a sensitivity of 0.750, a specificity of 0.863, a positive predictive value of 0.488, and an F1-score of 0.592. Formal comparisons did not demonstrate statistically significant AUC superiority over the other algorithms after Holm correction. Exploratory decision curve analysis showed a greater net benefit than treat-all and treat-none strategies across threshold probabilities of 0.06–0.35. ESR, age, CRP, and CRP-derived indices were the most influential model features. **Conclusions**: Routine laboratory data may provide adjunctive information for severity stratification, but the modest event count, limited positive predictive value, and absence of external validation preclude stand-alone clinical use.

## 1. Introduction

Acute appendicitis is the most common surgical emergency in children and a leading cause of pediatric abdominal pain, yet its clinical diagnosis and early severity stratification remain challenging [1,2]. Unlike adults, children often present with atypical, rapidly evolving, and nonspecific symptoms that overlap with other common childhood illnesses [3,4,5,6,7,8]. Younger children may be unable to articulate their symptoms clearly and may exhibit vague gastrointestinal manifestations, contributing to under-recognition and diagnostic delay. Delayed recognition increases the risk of perforation, abscess, and peritonitis. Clinicians must therefore maintain a high index of suspicion when evaluating children with otherwise unexplained abdominal pain.

By convention, appendicitis is classified as uncomplicated (UCA) or complicated (CA). Complicated appendicitis is characterized by perforation, gangrene, abscess, phlegmon, or diffuse peritonitis [5,9]. Early differentiation is clinically important: uncomplicated appendicitis is generally treated with timely appendectomy, and carefully selected patients may be considered for non-operative management [10]. In contrast, complicated appendicitis is associated with greater morbidity and more often requires urgent surgery, prolonged antibiotic therapy, drainage, longer hospitalization, and the management of postoperative complications [11]. Misclassification at presentation may therefore result either in unnecessary escalation for less severe disease or in a harmful delay for advanced disease.

Traditional diagnostic tools, including pediatric scoring systems, ultrasound, CT, and MRI, provide useful information but have limitations in children. The Alvarado score and Pediatric Appendicitis Score may perform less consistently in younger age groups, and ultrasound is operator-dependent. CT is highly sensitive but exposes children to ionizing radiation [12]. Routine laboratory markers also have limited standalone performance. White blood cell count is elevated in many causes of abdominal pain and is neither sufficiently sensitive nor specific for appendicitis [13]. C-reactive protein (CRP) may rise later in the inflammatory course, reducing its value in early presentations. No single clinical or laboratory feature therefore reliably distinguishes complicated from uncomplicated disease in all children.

Recently, attention has turned to composite inflammatory indices that combine multiple blood parameters. Ratios and indices such as the neutrophil-to-lymphocyte ratio (NLR), platelet-to-lymphocyte ratio (PLR), and systemic immune-inflammation index (SII) may reflect systemic inflammatory activity more comprehensively than individual measurements. A recent pediatric study, for example, reported potential diagnostic value for SII in acute appendicitis [14]. Such composite markers may also provide information about disease severity.

Machine learning (ML) offers a further advance by exploiting these multidimensional data patterns. Unlike conventional statistics, ML algorithms can learn complex, nonlinear relationships among routine lab values, vital signs, and demographics to distinguish UCA from CA. For example, a recent ML benchmarking study on pediatric appendicitis reported a Random Forest model achieving 98.1% accuracy for appendicitis diagnosis. In practice, ML can integrate dozens of admission lab variables simultaneously and uncover subtle cues that humans might miss [12]. Importantly, explainable AI tools such as Shapley Additive Explanations (SHAP) can attribute model predictions to individual features, helping clinicians understand why a case is flagged as high-risk.

We aimed to develop and internally validate machine-learning models for predicting complicated appendicitis in children using a compact set of routine admission laboratory parameters and age. Explainability analyses were applied to examine whether model behaviour was aligned with clinically plausible inflammatory patterns.

## 2. Materials and Methods

### 2.1. Study Design and Ethical Approval

This retrospective prediction-model development and internal validation study was conducted at a tertiary academic pediatric surgery centre. The study evaluated whether routinely available admission laboratory measurements could support early risk stratification for complicated appendicitis among children with surgically confirmed acute appendicitis. Accordingly, the target population consisted of patients with confirmed appendicitis, and the clinical objective was to distinguish complicated from uncomplicated disease using data available at emergency department presentation. The study was conducted in accordance with the Declaration of Helsinki and was approved by the Kocaeli University Non-Interventional Clinical Research Ethics Committee (KOU GOKAEK; protocol code GOKAEK-2026/02/38; approval date: 15 January 2026). Because of the retrospective design and anonymized data structure, informed consent was waived by the ethics committee.

### 2.2. Study Population and Outcome Definition

Children who underwent appendectomy for acute appendicitis between January 2020 and December 2024 were evaluated. Patients were classified according to operative and pathological disease severity. The primary outcome was complicated appendicitis, encoded as a binary endpoint. Complicated appendicitis was defined as appendicitis with perforation, gangrene, abscess, phlegmon, diffuse peritonitis, or comparable advanced inflammatory disease documented intraoperatively and/or pathologically [15]. Patients without these findings were classified as having uncomplicated appendicitis.

### 2.3. Predictor Set and Feature Engineering

Only age and laboratory parameters obtained at emergency department admission were used. Original variables included age in months, white blood cell count, neutrophil count, lymphocyte count, monocyte count, eosinophil count, basophil count, erythrocyte sedimentation rate, and C-reactive protein.

Six derived inflammatory indices were calculated from these admission variables: the neutrophil-to-lymphocyte ratio, monocyte-to-lymphocyte ratio, white blood cell-to-lymphocyte ratio, C-reactive protein-to-white blood cell ratio, C-reactive protein-to-lymphocyte ratio, and the C-reactive protein × neutrophil product. These indices were selected because they are biologically plausible, transparent, easily reproducible, and directly calculable from routine emergency laboratory tests.

### 2.4. Model Development Strategy

The analysis was planned as internal validation. The cohort was split into a stratified training set and an isolated stratified test set using a 70:30 ratio. Stratification preserved the prevalence of complicated appendicitis across the training and test partitions. The test set was reserved strictly for final model evaluation and was not used for hyperparameter tuning, feature selection, threshold selection, or model selection.

Five machine learning models were prespecified before test-set evaluation: elastic-net logistic regression, random forest, XGBoost, LightGBM, and CatBoost. These algorithms represented complementary model families, including a penalized linear baseline, a bagged tree ensemble, and gradient-boosting approaches. CatBoost was designated as the primary model before evaluation because it can model nonlinear relationships and interactions while supporting SHAP-based explanation. This designation was not based on achieving the highest observed test-set value for every performance metric.

Hyperparameter tuning was performed exclusively within the training set using stratified 5-fold cross-validation. Receiver operating characteristic area under the curve was used as the tuning objective.

Because complicated appendicitis represented the minority class, class weighting or equivalent positive-class weighting was used during model fitting. Additional predefined operating-point analyses were performed as supplementary robustness checks to evaluate whether alternative imbalance-aware strategies changed the sensitivity–specificity trade-off.

### 2.5. Model Performance Evaluation

Final model performance was evaluated once on the isolated test set. Discrimination was assessed using the receiver operating characteristic area under the curve and the precision-recall area under the curve. Precision-recall analysis was emphasized because the prevalence of complicated appendicitis was low, and precision-recall curves are more informative than ROC curves when evaluating minority-class performance.

At the default 0.50 probability threshold, sensitivity, specificity, positive predictive value, F1-score, Brier score, and confusion-matrix counts were calculated. Sensitivity was prioritized as a clinically important metric because false-negative classification of complicated appendicitis could delay the escalation of diagnostic or therapeutic management. Specificity and positive predictive value were interpreted alongside sensitivity to account for the clinical cost of false-positive high-risk classification.

Nonparametric bootstrap resampling with 1000 iterations was used to estimate 95% confidence intervals for key test-set performance metrics. The same isolated test set was used for all algorithms. Confidence intervals were reported to convey uncertainty, but overlap between intervals was not treated as a formal test of differences between correlated model performances.

Calibration was assessed descriptively using calibration curves, Brier scores, calibration intercepts, and calibration slopes. Because the study was an internal validation analysis, predicted probabilities were not considered ready for direct clinical decision-making without external or temporal validation and potential recalibration.

### 2.6. Formal Model Comparison and Decision Curve Analysis

Pairwise differences in test-set ROC AUC between the prespecified primary CatBoost model and each comparator were assessed using the correlated-sample DeLong method [16]. All comparisons used predictions from the same isolated test-set patients. Two-sided *p* values were adjusted across the four comparisons using the Holm method. These analyses tested for evidence of superiority and were not interpreted as equivalence tests. Decision curve analysis was used to quantify net benefit across threshold probabilities from 0.01 to 0.60 and compared each model with treat-all and treat-none strategies [17]. The test set was not used to select or optimize a clinical threshold; decision curve findings were treated as exploratory internal-validation evidence.

### 2.7. Explainability and Feature Importance

The primary CatBoost model was interpreted using SHAP values and permutation importance. Mean absolute SHAP values were used to quantify the average contribution of each feature to model output. Permutation importance was calculated as the decrease in test-set area under the curve after random perturbation of each feature. Using both approaches allowed assessment of whether model explanations were stable and clinically coherent across complementary importance methods.

SHAP values were interpreted as explanations of model behaviour rather than causal estimates or proof of independent biomarker effects. Feature-importance findings were evaluated in relation to descriptive statistics and univariate discrimination. Variables with weak standalone discrimination but nonzero model importance were interpreted cautiously and were not presented as primary biological findings. The final interpretation focused on systemic inflammatory burden, age, C-reactive protein-derived indices, and erythrocyte sedimentation rate.

### 2.8. Statistical Analysis

Continuous variables were summarized as medians and interquartile ranges. Between-group comparisons for uncomplicated and complicated appendicitis were performed using the Mann–Whitney U test. Benjamini–Hochberg false-discovery-rate-adjusted q-values were reported for multiple comparisons, and directional univariate AUC was calculated as a descriptive measure of standalone discrimination. The cohort included 93 complicated appendicitis events and 15 candidate predictors, corresponding to an approximate event-to-candidate-predictor ratio of 6.2. This ratio was treated as a descriptive indicator rather than a definitive adequacy rule because conventional events-per-variable guidance was developed primarily for regression modelling. All statistical and machine-learning analyses were conducted in Python 3.10 using pandas, NumPy, SciPy, scikit-learn, XGBoost, LightGBM, CatBoost, SHAP, and statsmodels.

## 3. Results

### 3.1. Cohort Characteristics and Univariate Discrimination

The final cohort included 628 children with surgically confirmed acute appendicitis, of whom 535 were classified as having uncomplicated appendicitis and 93 as having complicated appendicitis.

Baseline laboratory characteristics and univariate discriminatory performance are summarized in Table 1. The most consistent between-group differences were observed for inflammatory markers and CRP-derived composite indices. ESR was higher in children with complicated appendicitis than in those with uncomplicated appendicitis, with median values of 21.00 mm/h (IQR, 20.00–39.00) versus 12.00 mm/h (IQR, 6.00–19.50), respectively (*p* < 0.001). ESR also showed the highest standalone AUC among the evaluated predictors (AUC, 0.764). CRP demonstrated a similar pattern, with a median value of 79.35 mg/L (IQR, 30.50–157.62) in complicated appendicitis compared with 17.16 mg/L (IQR, 4.43–47.70) in uncomplicated appendicitis (*p* < 0.001; AUC, 0.739).

CRP-derived composite indices also showed significant univariate separation. The CRP/WBC ratio was higher in complicated appendicitis, with a median of 5.77 (IQR, 2.25–11.32) compared with 1.30 (IQR, 0.33–3.43) in uncomplicated appendicitis (*p* < 0.001; AUC, 0.742). The CRP/lymphocyte ratio was likewise higher in complicated appendicitis, with median values of 43.69 (IQR, 10.50–92.51) versus 9.64 (IQR, 2.61–26.15), respectively (*p* < 0.001; AUC, 0.705). The CRP × neutrophil product was also significantly increased in complicated disease, with a median value of 677.28 (IQR, 165.83–2064.66) compared with 153.59 (IQR, 40.22–489.43) in uncomplicated appendicitis (*p* < 0.001; AUC, 0.688).

Children with complicated appendicitis were younger, with a median age of 92.00 months (IQR, 58.00–153.00), compared with 134.00 months (IQR, 104.50–174.00) in the uncomplicated group (*p* < 0.001; AUC, 0.657). In contrast, individual leukocyte parameters showed limited standalone separation. WBC, neutrophil count, lymphocyte count, monocyte count, eosinophil count, and basophil count were not significantly different between groups after false-discovery-rate adjustment. Similarly, the NLR, MLR, and WBC/lymphocyte ratio showed weak univariate discrimination, with directional AUC values close to 0.50. These findings suggested that systemic inflammatory burden, particularly ESR, CRP, and CRP-derived composite markers, carried more severity-related information than isolated leukocyte counts alone.

### 3.2. Test-Set Model Performance

**Figure 1:**
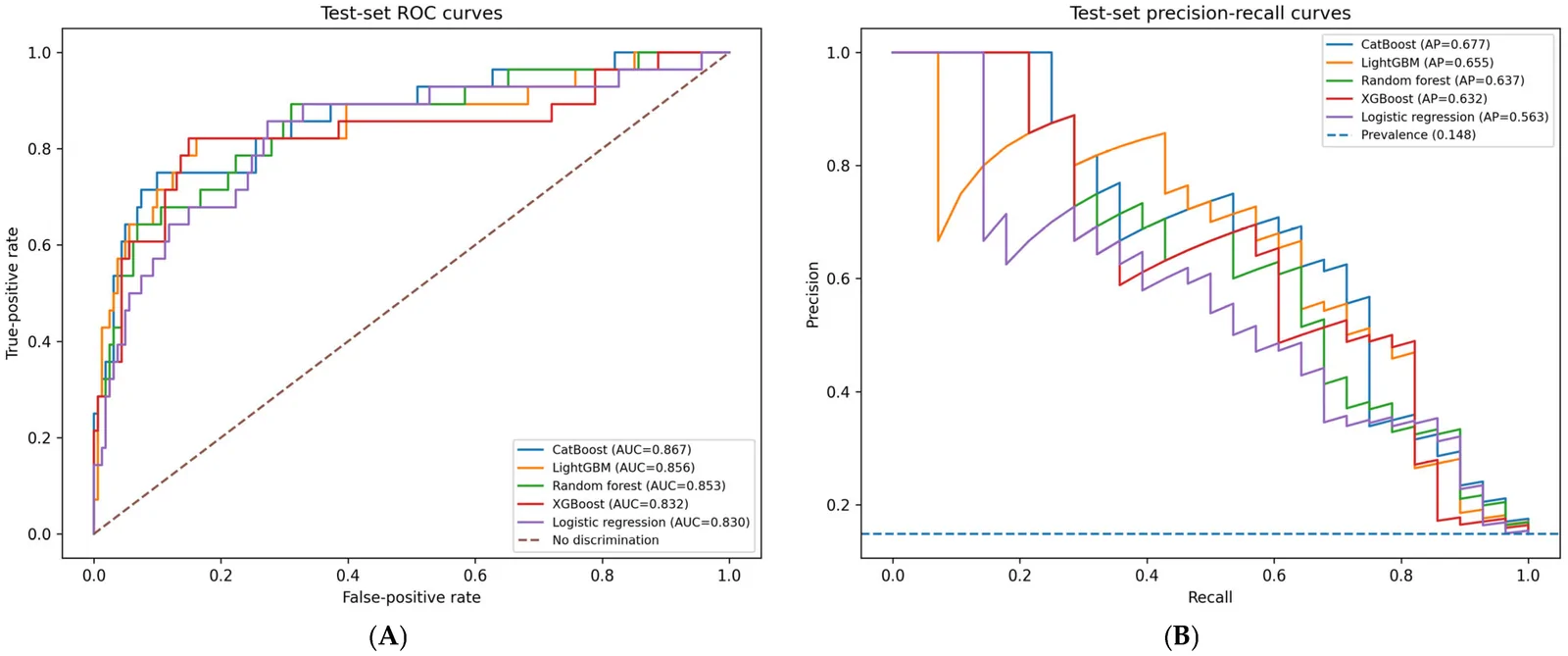
Test-set discrimination of the five prespecified machine-learning models. (**A**) shows ROC curves. (**B**) shows precision-recall curves. Values in legends are test-set ROC AUC or average precision.

The discrimination curves for the five prespecified models are shown in Figure 1, and detailed test-set performance metrics are presented in Table 2. All models were developed exclusively within the training set and evaluated once on the isolated test set. Test-set ROC AUC values ranged from 0.830 for elastic-net logistic regression to 0.867 for CatBoost. Formal paired comparisons are reported separately below; small numerical differences in point estimates were not interpreted as evidence of algorithmic superiority.

The prespecified primary CatBoost model achieved a test ROC AUC of 0.867 (95% CI, 0.782–0.938) and a precision-recall AUC of 0.677 (95% CI, 0.509–0.825). At the default 0.50 probability threshold, CatBoost yielded a sensitivity of 0.750 (95% CI, 0.577–0.897), a specificity of 0.863 (95% CI, 0.816–0.915), a positive predictive value of 0.488 (95% CI, 0.349–0.633), and an F1-score of 0.592 (95% CI, 0.449–0.712). The confusion matrix included 139 true negatives, 22 false positives, 7 false negatives, and 21 true positives. The Brier score was 0.117.

LightGBM showed comparable discrimination, with a test ROC AUC of 0.856 (95% CI, 0.756–0.938) and a precision-recall AUC of 0.655 (95% CI, 0.486–0.822). At the default threshold, LightGBM yielded a sensitivity of 0.607, a specificity of 0.944, a positive predictive value of 0.654, and the highest F1-score among the five models (0.630). Its Brier score was 0.085. CatBoost nevertheless remained the primary model because that designation had been prespecified before test-set evaluation.

Random Forest achieved a test ROC AUC of 0.853 (95% CI, 0.761–0.925) and a precision-recall AUC of 0.637 (95% CI, 0.466–0.796), with a sensitivity of 0.679 and a specificity of 0.888. XGBoost achieved a test ROC AUC of 0.832 (95% CI, 0.713–0.925) and a precision-recall AUC of 0.632 (95% CI, 0.450–0.791), with a sensitivity of 0.607 and a specificity of 0.925. Elastic-net logistic regression achieved a test ROC AUC of 0.830 (95% CI, 0.732–0.913), a precision-recall AUC of 0.563 (95% CI, 0.394–0.732), a sensitivity of 0.679, and a specificity of 0.839. Supplementary sensitivity analyses for class imbalance and operating-threshold choice are presented in Table S1, and calibration curves for the five prespecified models are shown in Figure S1.

### 3.3. Formal Model Comparisons and Decision Curve Analysis

**Figure 2:**
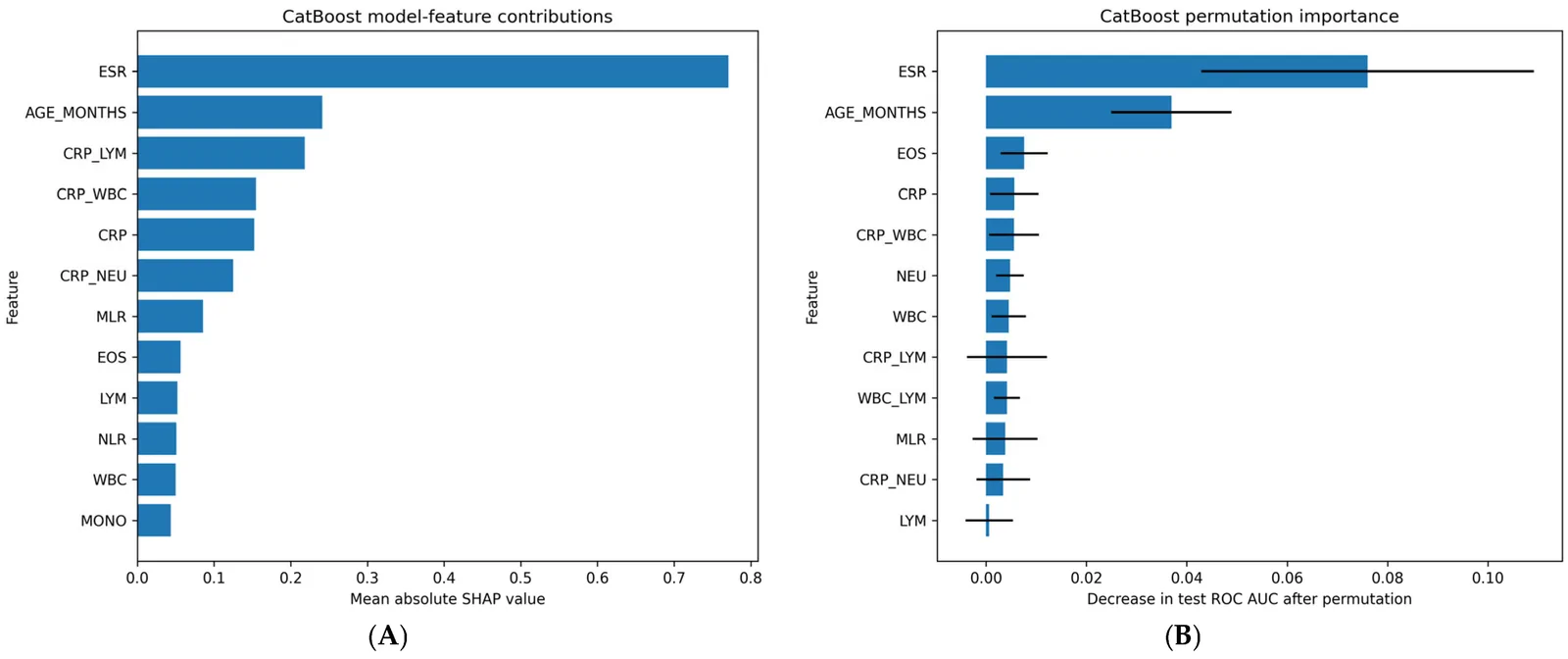
Feature importance for the prespecified primary CatBoost model. (**A**) shows mean absolute SHAP values. (**B**) shows permutation-based test-AUC decrease. Values represent model-feature contributions and should not be interpreted as biological or causal effects.

Paired DeLong comparisons did not demonstrate statistically significant superiority of CatBoost over LightGBM (AUC difference, 0.011; 95% CI, −0.047 to 0.068), Random Forest (difference, 0.014; 95% CI, −0.016 to 0.045), XGBoost (difference, 0.035; 95% CI, −0.022 to 0.092), or elastic-net logistic regression (difference, 0.037; 95% CI, −0.021 to 0.095); all Holm-adjusted *p* values were 0.832 (Table 3). Decision curve analysis showed that CatBoost provided a greater net benefit than both treat-all and treat-none strategies across the broad threshold-probability range of 0.06–0.35 (Figure 2). Small isolated positive-net-benefit segments at higher thresholds were not emphasized because of instability in a test set containing only 28 complicated cases.

### 3.4. Feature Importance and Model Explainability

**Figure 3:**
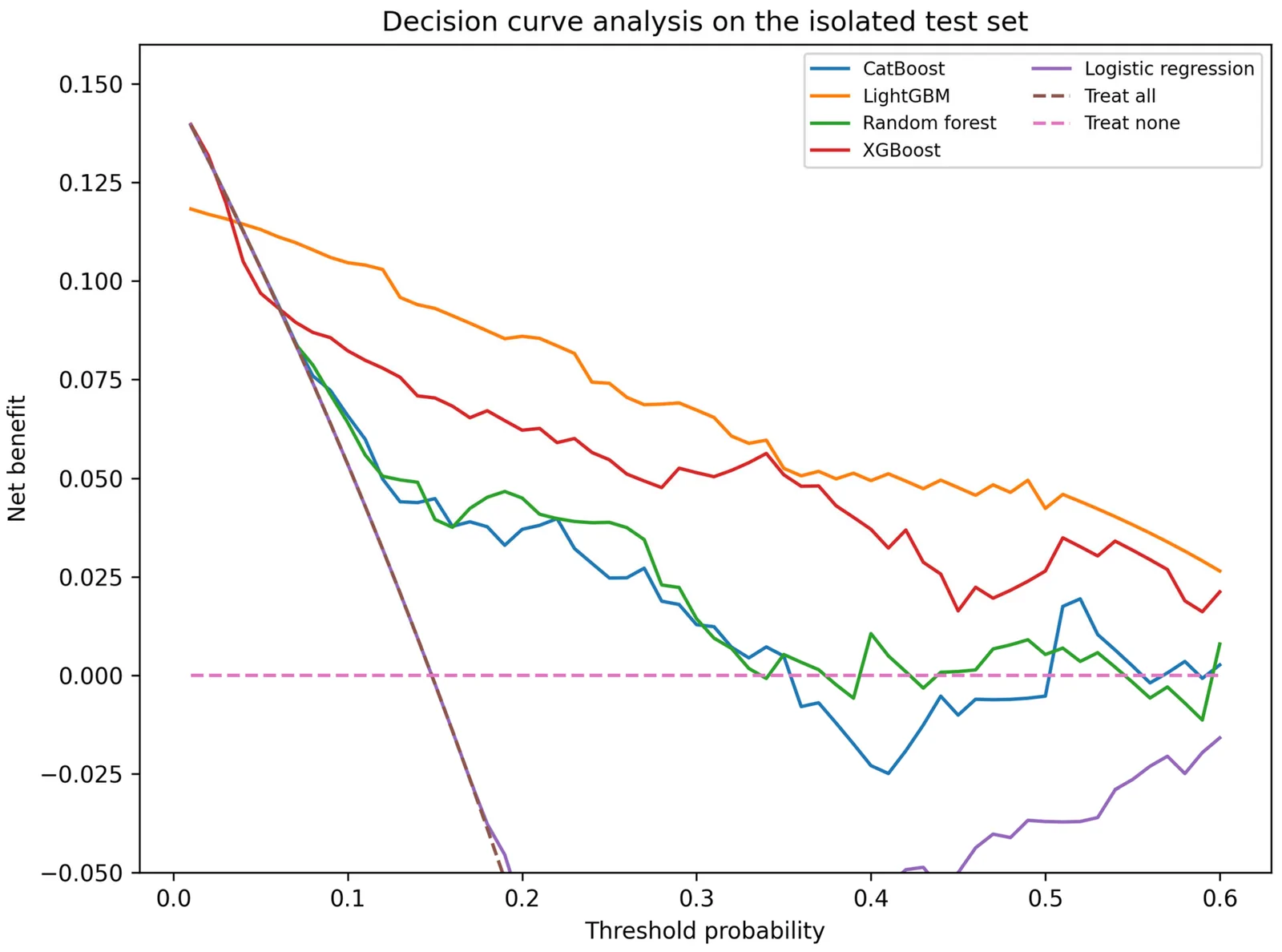
Decision curve analysis for the five prespecified models on the isolated test set. Net benefit is shown across threshold probabilities from 0.01 to 0.60 together with treat-all and treat-none strategies. The analysis is exploratory and was not used to select a deployment threshold.

Feature importance for the primary CatBoost model is shown in Figure 3. Mean absolute SHAP values identified ESR, age, CRP/lymphocyte ratio, CRP/WBC ratio, CRP, and the CRP x neutrophil product as the most influential model features. Permutation importance provided a complementary assessment, with ESR and age producing the largest decreases in test-set AUC after permutation. These findings describe how the fitted model generated predictions; they do not establish biological importance, causality, or independent prognostic effects.

## 4. Discussion

In this retrospective prediction-model development and internal validation study, a compact set of routine admission laboratory parameters and age provided a moderate-to-good severity signal among children with surgically confirmed appendicitis. ESR, CRP, CRP-derived indices, and younger age showed the most consistent univariate separation. The prespecified CatBoost model achieved a test ROC AUC of 0.867, but paired DeLong testing did not demonstrate statistically significant superiority over the other algorithms. Decision curve analysis suggested a potential net benefit over treat-all and treat-none strategies across thresholds of 0.06–0.35. Model-explanation analyses identified ESR, age, CRP, and CRP-derived indices as influential features in the fitted model.

Distinguishing uncomplicated from complicated appendicitis is clinically important because these entities do not represent merely different points on a laboratory spectrum; they often imply different risks, treatment priorities, and resource needs. Uncomplicated appendicitis is usually managed successfully with timely appendectomy, and selected cases may even be considered for non-operative treatment under appropriate clinical conditions [10]. In contrast, complicated appendicitis reflects advanced local or systemic inflammation, including perforation, gangrene, abscess, phlegmon, or diffuse peritonitis, and is more often associated with prolonged antibiotic therapy, drainage procedures, urgent operative decision-making, longer hospitalization, and postoperative morbidity. Therefore, early severity stratification has a dual clinical purpose: to avoid under-recognition of children with advanced disease, while also reducing the risk of overtreatment or unnecessary escalation in children with less severe inflammation.

This balance is particularly relevant in pediatric patients, in whom symptoms may be nonspecific and diagnostic delays remain common, especially at younger ages [18,19]. A child with unrecognized complicated appendicitis may deteriorate rapidly or present late with abscess, sepsis, or prolonged recovery. Conversely, treating every child as high risk may increase imaging, radiation exposure, antibiotic use, admission burden, and procedural intensity without clear benefit. Our finding that younger children had a higher risk of complicated disease is consistent with this clinical pattern. Rather than suggesting that age alone is sufficient for decision-making, our results indicate that age may provide useful contextual information when interpreted together with inflammatory markers obtained at admission. The strongest univariate signals in the present cohort were observed for ESR, CRP, and CRP-derived indices. This finding is biologically plausible, as complicated appendicitis represents a more advanced inflammatory state than uncomplicated disease. CRP has long been associated with disease severity and perforation risk in pediatric appendicitis, although its performance as an isolated marker is insufficient for definitive classification [20,21]. In our analysis, CRP-derived indices such as the CRP/WBC ratio, CRP/lymphocyte ratio, and the CRP × neutrophil product showed stronger descriptive separation than several isolated leukocyte parameters. This supports the concept that combined inflammatory markers may better reflect systemic inflammatory burden than single blood-count components alone [14,22]. However, these indices should not be interpreted as independent diagnostic tests; rather, they contributed to a multivariable risk-stratification framework.

The machine-learning results should be interpreted conservatively. CatBoost achieved the highest ROC AUC, whereas LightGBM achieved the highest F1-score at the default threshold. CatBoost remained the primary model because it had been designated before test-set evaluation, not because it was statistically superior or uniformly best across all metrics. Formal paired DeLong comparisons did not demonstrate significant AUC differences after Holm correction. These results indicate that the predictive signal was shared across several modelling families and do not justify promoting one algorithm as definitively superior.

Because complicated appendicitis represented a minority outcome, clinical interpretation cannot rely on ROC AUC alone. At the default threshold, CatBoost sensitivity was 0.750 and positive predictive value was 0.488; thus, approximately half of the children flagged as high risk did not have complicated disease. A positive output should therefore not independently trigger imaging, antibiotics, admission, drainage, or surgery. Its plausible role is limited to adjunctive risk stratification that may prompt closer reassessment or senior surgical review when interpreted alongside symptoms, examination findings, imaging, and clinical judgement. Decision curve analysis provided exploratory support for net benefit across thresholds of 0.06–0.35, but it was derived from the same internal test set and does not establish a clinically appropriate deployment threshold.

SHAP and permutation analyses were used to explain the fitted CatBoost model, not to identify causal biomarkers. ESR, age, CRP/lymphocyte ratio, CRP/WBC ratio, CRP, and the CRP x neutrophil product were influential model features, and ESR and age had the largest permutation effects. These rankings indicate how the model’s predictions changed when features varied or were perturbed. They do not demonstrate biological importance, independent prognostic value, or causal relationships. The terminology throughout the manuscript was revised accordingly.

The laboratory-only design was deliberate. Age and admission laboratory measurements are objective, routinely available, and reproducible, allowing evaluation of a compact early severity signal. The study was not intended to represent the complete clinical pathway. Symptom duration, body temperature, abdominal findings, and imaging were not uniformly available and therefore could not be incorporated or used to reconstruct the Alvarado score or Pediatric Appendicitis Score. The findings should not be interpreted as evidence of superiority over conventional scores. Future multimodal studies should quantify the incremental value of laboratory-based machine learning beyond clinical assessment and imaging.

Several limitations should be acknowledged. First, this was a retrospective single-centre study, and the reported performance represents internal validation only. Even with an isolated test set, performance may decline at institutions with different referral patterns, laboratory platforms, missing-data processes, disease prevalence, and treatment pathways. Second, 93 events were available for 15 candidate predictors, yielding an approximate event-to-candidate-predictor ratio of 6.2; this modest event count may limit the stability of flexible models. Third, the predictor set excluded potentially informative symptoms, physical findings, vital signs, and imaging. Fourth, positive predictive value was limited, and decision-curve findings were exploratory rather than evidence of clinical effectiveness. Fifth, calibration and threshold behaviour require external assessment and potential recalibration. Multicentre or temporal validation and prospective evaluation of downstream clinical consequences are required before implementation.

## 5. Conclusions

In summary, routine admission laboratory data and age contained an internally reproducible severity signal for complicated pediatric appendicitis. CatBoost showed good discrimination, but formal comparisons did not establish superiority over the other prespecified algorithms, and its positive predictive value remained limited. The model should be considered an exploratory adjunct to, rather than a replacement for, clinical assessment, imaging, and surgical judgement. External validation, recalibration, and prospective evaluation of clinical consequences are required before use in patient care.

## Figures and Tables

**Table 1:** Baseline laboratory characteristics and univariate discrimination. Continuous variables are shown as median (IQR). *p* values are from Mann–Whitney U tests; q values are Benjamini–Hochberg adjusted.

Variable	UCA Median (IQR)	CA Median (IQR)	*p* Value	q Value	AUC
WBC	14.25 (10.83–17.10)	13.74 (9.43–17.09)	0.603	0.650	0.517
Neutrophils	10.90 (7.53–14.16)	9.96 (6.16–14.60)	0.307	0.538	0.533
Lymphocytes	1.76 (1.20–2.45)	1.84 (1.20–2.68)	0.216	0.462	0.54
Monocytes	0.90 (0.67–1.19)	0.96 (0.60–1.26)	0.645	0.650	0.515
Eosinophils	0.04 (0.01–0.10)	0.04 (0.01–0.15)	0.615	0.650	0.516
Basophils	0.04 (0.02–0.05)	0.04 (0.02–0.06)	0.650	0.650	0.515
ESR	12.00 (6.00–19.50)	21.00 (20.00–39.00)	<0.001	<0.001	0.764
CRP	17.16 (4.43–47.70)	79.35 (30.50–157.62)	<0.001	<0.001	0.739
Age (months)	134.00 (104.50–174.00)	92.00 (58.00–153.00)	<0.001	<0.001	0.657
NLR	6.22 (3.51–10.12)	6.28 (2.25–10.13)	0.323	0.538	0.532
MLR	0.51 (0.33–0.74)	0.50 (0.27–0.77)	0.500	0.650	0.522
WBC/LYM	7.97 (4.93–12.01)	8.18 (3.59–11.62)	0.383	0.575	0.528
CRP/WBC	1.30 (0.33–3.43)	5.77 (2.25–11.32)	<0.001	<0.001	0.742
CRP/LYM	9.64 (2.61–26.15)	43.69 (10.50–92.51)	<0.001	<0.001	0.705
CRP x NEU	153.59 (40.22–489.43)	677.28 (165.83–2064.66)	<0.001	<0.001	0.688

**Table 2:** Isolated test-set performance of five prespecified models. Models were tuned only within the training set by stratified 5-fold cross-validation. Values in parentheses are bootstrap 95% confidence intervals.

Model	Training CV ROC AUC	ROC AUC	PR AUC	Sensitivity	Specificity	PPV	F1	Brier	TN	FP	FN	TP
CatBoost	0.827	0.867 (0.782–0.938)	0.677 (0.509–0.825)	0.750 (0.577–0.897)	0.863 (0.816–0.915)	0.488 (0.349–0.633)	0.592 (0.449–0.712)	0.117	139	22	7	21
LightGBM	0.826	0.856 (0.756–0.938)	0.655 (0.486–0.822)	0.607 (0.429–0.790)	0.944 (0.908–0.976)	0.654 (0.474–0.826)	0.630 (0.464–0.761)	0.085	152	9	11	17
Random forest	0.834	0.853 (0.761–0.925)	0.637 (0.466–0.796)	0.679 (0.500–0.840)	0.888 (0.841–0.933)	0.514 (0.355–0.667)	0.585 (0.437–0.711)	0.115	143	18	9	19
XGBoost	0.834	0.832 (0.713–0.925)	0.632 (0.450–0.791)	0.607 (0.417–0.778)	0.925 (0.886–0.963)	0.586 (0.412–0.759)	0.596 (0.431–0.731)	0.097	149	12	11	17
Logistic regression	0.798	0.830 (0.732–0.913)	0.563 (0.394–0.732)	0.679 (0.514–0.844)	0.839 (0.782–0.892)	0.422 (0.293–0.571)	0.521 (0.379–0.649)	0.163	135	26	9	19

**Table 3:** Pairwise DeLong comparisons of test-set ROC AUC with CatBoost as the prespecified primary model. AUC differences are CatBoost minus comparator. Two-sided *p* values were adjusted across four comparisons using the Holm method. Nonsignificant results indicate that superiority was not demonstrated; they do not establish equivalence.

Comparator	CatBoost AUC	Comparator AUC	AUC Difference	95% CI of Difference	Raw *p*	Holm-Adjusted *p*
LightGBM	0.867	0.856	0.011	−0.047 to 0.068	0.711	0.832
Random forest	0.867	0.853	0.014	−0.016 to 0.045	0.352	0.832
XGBoost	0.867	0.832	0.035	−0.022 to 0.092	0.227	0.832
Logistic regression	0.867	0.830	0.037	−0.021 to 0.095	0.208	0.832

## Data Availability

The data presented in this study are not publicly available due to privacy and ethical restrictions. De-identified data may be made available from the corresponding author upon reasonable request and subject to Institutional Review Board (Ethics Committee) approval and applicable data-sharing agreements.

## Supplementary Materials

The following supporting information can be downloaded at: https://www.mdpi.com/article/10.3390/diagnostics16162644/s1, Table S1: Sensitivity analysis for class imbalance and operating-threshold choice. Figure S1: Calibration curves for the five prespecified models on the isolated test set. Observed event proportions are plotted against mean predicted probabilities; the diagonal line represents perfect calibration. The curves provide descriptive internal-validation information and should not be interpreted as evidence of external calibration.

## Abbreviations

The following abbreviations are used in this manuscript:

AUC	Area under the curve
BASO	Basophil count
CA	Complicated appendicitis
CI	Confidence interval
CRP	C-reactive protein
CRP/LYM	C-reactive protein-to-lymphocyte ratio
CRP/WBC	C-reactive protein-to-white blood cell ratio
DCA	Decision curve analysis
EOS	Eosinophil count
EPV	Events per variable
ESR	Erythrocyte sedimentation rate
IQR	Interquartile range
LightGBM	Light Gradient Boosting Machine
LYM	Lymphocyte count
ML	Machine learning
MLR	Monocyte-to-lymphocyte ratio
MONO	Monocyte count
NEU	Neutrophil count
NLR	Neutrophil-to-lymphocyte ratio
PAS	Pediatric Appendicitis Score
PLR	Platelet-to-lymphocyte ratio
PPV	Positive predictive value
PR AUC	Precision-recall area under the curve
ROC AUC	Receiver operating characteristic area under the curve
SHAP	Shapley Additive Explanations
SII	Systemic immune-inflammation index
SMOTE	Synthetic Minority Oversampling Technique
UCA	Uncomplicated appendicitis
WBC	White blood cell count
WBC/LYM	White blood cell-to-lymphocyte ratio
XGBoost	Extreme Gradient Boosting
