# Supplementary material for: Predicting Complicated Appendicitis: What Can Machine Learning Add?
Source: Diagnostics (Basel). 2026 Aug 19;16(16):2644. doi: 10.3390/diagnostics16162644 (PMC13511705; doi:10.3390/diagnostics16162644)
Supplement: Supplementary file 1 [file diagnostics-16-02644-s001.zip › diagnostics-4433615-supplementary.pdf]

Supplementary Materials

Table S1. Sensitivity analysis for class imbalance and operating-threshold choice.

Sensitivity analysis strategy	Threshold	ROC AUC	PR AUC	Sensitivity	Specificity	PPV	F1	Brier	TN	FP	FN	TP
Unweighted CatBoost (default)	0.50	0.876	0.752	0.464	1.000	1.000	0.634	0.072	161	0	15	13
Class-weighted CatBoost (primary)	0.50	0.867	0.677	0.750	0.863	0.488	0.592	0.117	139	22	7	21
Class-weighted CatBoost (F1-optimized CV)	0.51	0.867	0.677	0.750	0.894	0.553	0.636	0.117	144	17	7	21
Class-weighted CatBoost (sensitivity-prioritized CV)	0.44	0.867	0.677	0.750	0.826	0.429	0.545	0.117	133	28	7	21
SMOTE CatBoost (default)	0.50	0.876	0.665	0.750	0.851	0.467	0.575	0.117	137	24	7	21

All variants used the same prespecified simplified feature set and the same isolated test set. Thresholds were selected from training-set cross-validated predictions before test-set application.

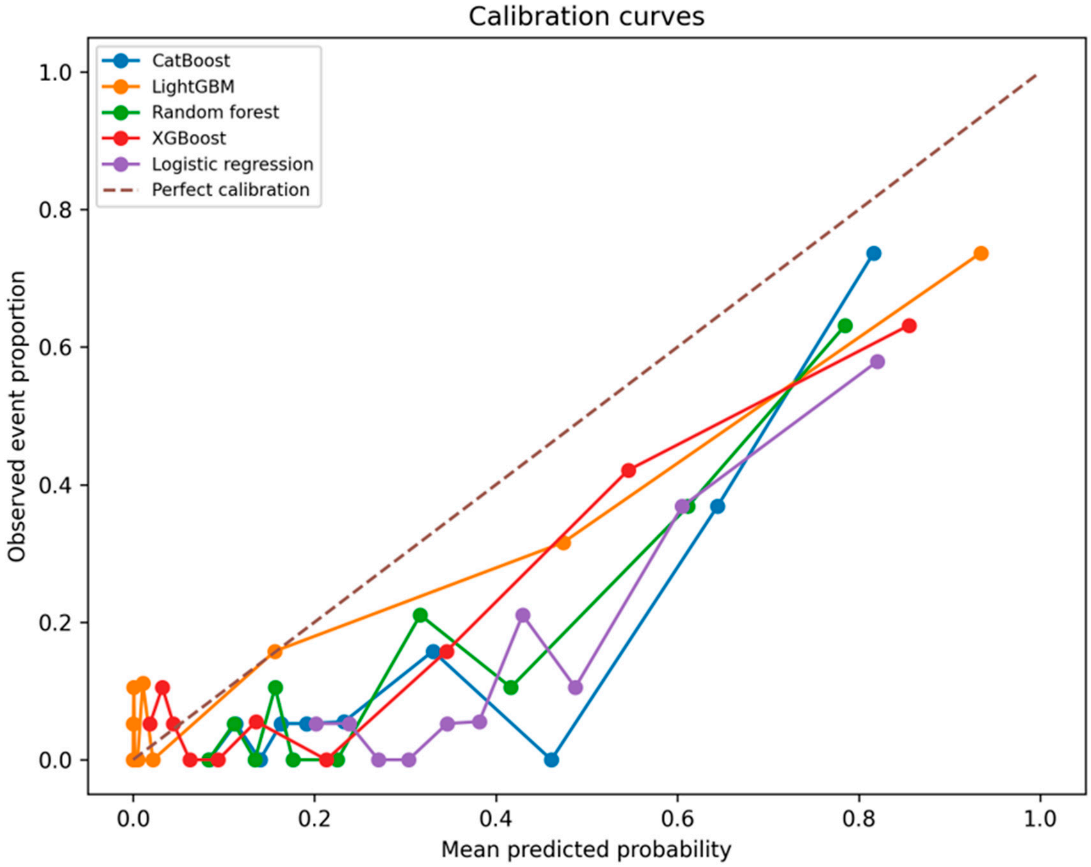

Figure S1. Calibration curves for the five prespecified models on the isolated test set. Observed event proportions are plotted against mean predicted probabilities; the diagonal line represents perfect calibration. The curves provide descriptive internal-validation information and should not be interpreted as evidence of external calibration.
